# Microstructure Evolution with Rapid Thermal Annealing Time in (001)-Oriented Piezoelectric PZT Films Integrated on (111) Si

**DOI:** 10.3390/ma16052068

**Published:** 2023-03-02

**Authors:** Yingying Wang, Hanfei Zhu, Yinxiu Xue, Peng Yan, Jun Ouyang

**Affiliations:** 1 Key Laboratory for Liquid-Solid Structure Evolution and Processing of Materials (Ministry of Education), School of Materials Science and Engineering, Shandong University, Jinan 250061, China; 2 Institute of Advanced Energy Materials and Chemistry, School of Chemistry and Chemical Engineering, Qilu University of Technology (Shandong Academy of Sciences), Jinan 250353, China; 3 Key Laboratory of High-efficiency and Clean Mechanical Manufacturing (Ministry of Education), School of Mechanical Engineering, Shandong University, Jinan 250061, China; 4 School of Materials Science and Engineering, Xiangtan University, Xiangtan 411105, China

**Keywords:** lead zirconate titanate (PZT), piezoelectric, micro-electro-mechanical systems (MEMS), Si, ferroelectric film, rapid thermal annealing (RTA), nanopores

## Abstract

In our recently published paper (Y.-Y. Wang et al., High performance LaNiO_3_-buffered, (001)-oriented PZT piezoelectric films integrated on (111) Si, *Appl. Phys. Lett.* 121, 182902, 2022), highly (001)-oriented PZT films with a large transverse piezoelectric coefficient *e_31,f_* prepared on (111) Si substrates were reported. This work is beneficial for the development of piezoelectric micro-electro-mechanical systems (Piezo-MEMS) because of (111) Si’s isotropic mechanical properties and desirable etching characteristics. However, the underlying mechanism for the achievement of a high piezoelectric performance in these PZT films going through a rapid thermal annealing process has not been thoroughly analyzed. In this work, we present complete sets of data in microstructure (XRD, SEM and TEM) and electrical properties (ferroelectric, dielectric and piezoelectric) for these films with typical annealing times of 2, 5, 10 and 15 min. Through data analyses, we revealed competing effects in tuning the electrical properties of these PZT films, i.e., the removal of residual PbO and proliferation of nanopores with an increasing annealing time. The latter turned out to be the dominating factor for a deteriorated piezoelectric performance. Therefore, the PZT film with the shortest annealing time of 2 min showed the largest *e_31,f_* piezoelectric coefficient. Furthermore, the performance degradation occurred in the PZT film annealed for 10 min can be explained by a film morphology change, which involved not only the change in grain shape, but also the generation of a large amount of nanopores near its bottom interface.

## 1. Introduction

Lead zirconate titanate (PZT) ferroelectric films have prominent applications in Si-based microelectronics because of their excellent ferroelectric, piezoelectric, dielectric and pyroelectric properties [1,2,3]. These applications include non-volatile ferroelectric random access memories, micro-energy harvesters, micro-actuators and sensors, etc. [1,2,4,5,6]. For PZT films with a chemical composition at the morphotropic phase boundary (MPB), a (001)-oriented film is generally considered to have the optimal piezoelectric and ferroelectric properties [7,8].

In our previous work, we successfully prepared highly (001)-oriented PZT thick films on (111) Si with an excellent piezoelectric performance by using a “two-step” method, i.e., a low-temperature magnetron sputtering deposition (350 °C) combined with a high temperature rapid thermal annealing (RTA) process [9]. This work is important because of the special characteristics of the (111) Si substrate. Not only does a (111) Si have isotropic mechanical properties (Young’s modulus, shear modulus and Poisson’s ratio) desirable for the fabrication of MEMS devices working in different motion modes [10], but it also shows steep and sharp etched edges ideal for high aspect-ratio MEMS structures [11]. The combination of a large piezoelectricity in (001) PZT with a good patternability of (111) Si opens up possibilities for the creation and application of novel piezo-MEMS devices. It is noted that, by using conductive LaNiO_3_ as the buffer layer and Pt/Ti as the diffusion barrier/bottom electrode layer, as well as a SiO_2_/(111) Si substrate, the interface diffusion and chemical reaction between the PZT layer and the (111) Si substrate were suppressed [12,13]. Furthermore, it was revealed that increasing the annealing time will create two competing effects on the electrical properties of the PZT films, i.e., the removal of residual PbO vs. proliferation of nanopores. However, in this letter, the microstructure and electrical properties of PZT films on (111) Si with an annealing time longer than 5 min were not analyzed, and hence, the two competing effects have not been fully explained.

Pores are very common in sintered ferroelectric ceramics, and they are often considered as an internal defect. Ferroelectric ceramics with a large amount of pores will display reduced spontaneous electric polarizations *(P_s_*, *P_r_*), coercive field (*E_c_*) and piezoelectric coefficients (*e_31_*/*d_31_*, *d_33_*), when compared with their dense counterparts [14,15,16,17]. On the other hand, because of a decreased dielectric constant (*ε_r_*), the piezoelectric voltage constant (*g_ij_*) and figure of merit (FOM = *g_ij_* · *d_ij_*) of a porous ferroelectric ceramic can be improved against its dense counterpart, which has been experimentally verified [18,19]. For a ferroelectric film, pores have similar effects on its electrical properties, as reported by Stancu et al. on porous PZT films [20]. Furthermore, due to their advantages in acoustic impedance matching with air or water [21,22,23] and in enhancing cell infiltration and tissue regeneration in biological systems [24,25], porous ferroelectrics, including both bulk ceramics and films, will fit in a number of applications in ambient transducers and medical implants.

In this work, the microstructure and electrical properties of the PZT films sputtered on (111) Si and annealed for different time periods (2 min, 5 min, 10 min and 15 min) were systematically analyzed. We revealed the evolution of the film’s microstructure with an annealing time, i.e., from the columnar grains to equiaxial grains, and the accompanied changes in their electrical properties. Specifically, the amount of nanopores and their effect on the transverse piezoelectric property of the films were analyzed.

## 2. Experimental

**Figure 1:**
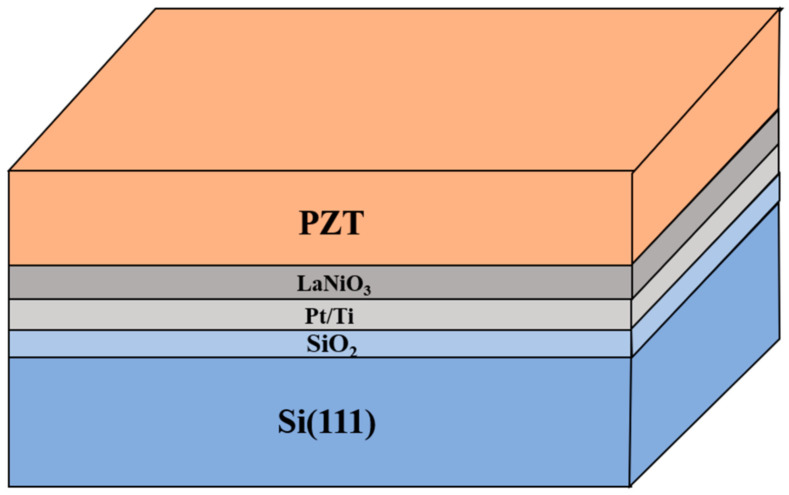
Diagram of the PZT/LaNiO_3_/Pt/Ti/SiO_2_/(111)Si heterostructure.

PZT films with a thickness of ~1.5 μm and a Zr/Ti ratio of 53/47 were deposited on a LaNiO_3_-buffered Pt/Ti/SiO_2_/(111)Si substrate in a multi-target RF magnetron sputtering system at a low temperature of 350 °C. The Pt/Ti bottom electrode with a thickness of about 130 nm was deposited at 300 °C on SiO_2_/(111)Si in a pure argon atmosphere. Then, a LaNiO_3_ layer of ~100 nm thick was sputtered at 350 °C, the same deposition temperature as that of the PZT film. Both the PZT film and the LaNiO_3_ buffer layer were deposited in an Ar/O_2_ mixed atmosphere with a 4:1 flow ratio, and their sputtering pressures were 1.2 Pa and 0.3 Pa, respectively. It is noted that, to compensate the volatilization loss of Pb during the deposition process, a 20% excessive amount of PbO was intentionally added to the raw materials for sintering of the PZT ceramic target [26,27]. A pure oxygen atmosphere is used for the cooling process to suppress the formation of oxygen vacancies. Lastly, during the RTA process using a previously reported recipe [26], the PZT films were kept at 700 °C for 2–15 min in a rapid thermal annealing furnace continuously fed with oxygen and were then naturally cooled down to room temperature [9]. A diagram of the PZT/LaNiO_3_/ Pt/Ti/SiO_2_/(111)Si heterostructure is depicted in Figure 1.

The phase structures and crystallographic orientations of the PZT films were detected by using XRD *θ*-2*θ* scans (Rigaku Dmax-2500 PC XRD diffractometer equipped with a Ni-filtered Cu K𝛼 radiation source, Tokyo, Japan). Nanostructures of the PZT films were investigated via scanning electron microscopy (SEM, Sigma 300, Zeiss, Oberkochen, Germany) and transmission electron microscopy (TEM, JEM-2010, JEOL, Tokyo, Japan), respectively. Cross-sectional SEM images of the PZT films were compared head to head, from which the grain morphology and its evolution with the annealing time were analyzed. The TEM analysis, on the other hand, revealed the nanopore morphology of the PZT films, as well as their out-of-plane and in-plane lattice parameters.

Room-temperature ferroelectric hysteresis loops (P-E loop, @10 kHz) and dielectric properties (C-V and C-*f* curves) were measured by using a Radiant Precision Premium ferroelectric tester (Radiant Technology, Albuquerque, NM, USA) and a TH2838H precision LCR meter (Tonghui Electronics, Changzhou, China), respectively. In the piezoelectric measurement, an AT100 laser Doppler vibrometer (Graphtec, Yokohama, Japan) was used to record tip displacements of PZT cantilever beams (20 mm × 3 mm × 0.5 mm) diced from the film samples (20 mm × 10 mm × 0.5 mm). These displacements were driven by an external sine wave AC voltage, from which the transverse piezoelectric coefficients *e_31,f_* were calculated. The AC frequency used for the piezoelectric measurement is 500 Hz, which is much lower than the resonant frequency of these PZT cantilever beams (~1700–1800 Hz) [9]. The deposition of dot-shaped Au top electrodes for the ferroelectric and dielectric tests, and rectangular-shaped Pt top electrodes for the piezoelectric measurement, were described in detail in Ref. [26].

## 3. Results and Discussion

**Figure 2:**
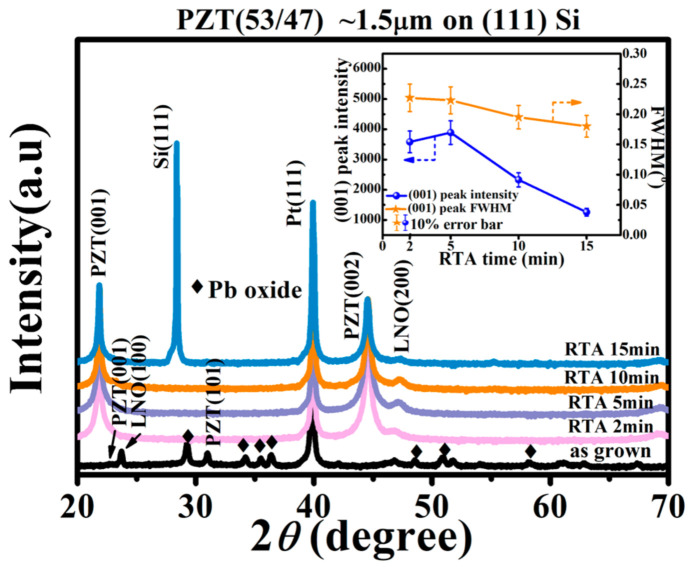
XRD *θ*-2*θ* scan patterns of the PZT films deposited on (111) Si before and after the RTA process with different annealing times. The inset shows the (001) PZT peak intensity and full width at half maximum (FWHM) as functions of the annealing time.

**Figure 3:**
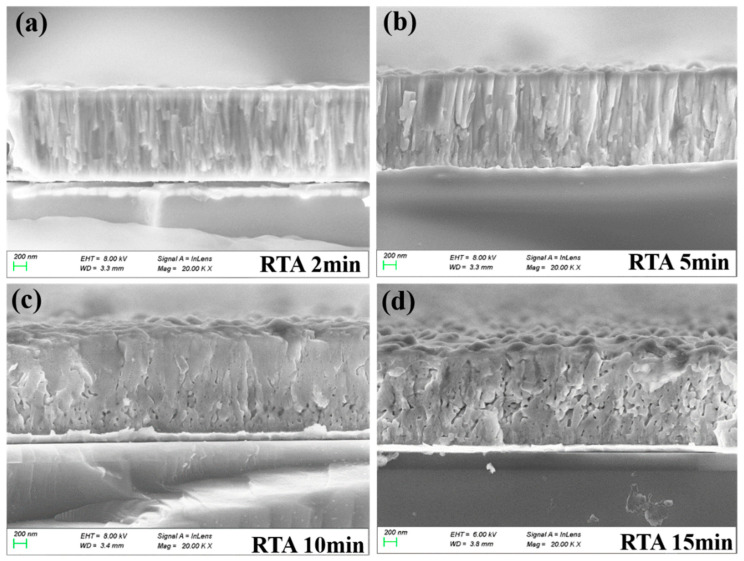
(**a**–**d**) Cross-sectional SEM images of PZT films deposited on (111) Si and annealed for 2 min, 5 min, 10 min and 15 min via a rapid thermal annealing process.

Figure 2 shows the diffraction patterns from the XRD θ-2θ scans of the as-grown PZT film and those underwent the rapid thermal annealing for different times (2–15 min). The as-grown film showed a weak crystallization of the perovskite PZT and a sizable amount of residual PbO. The existence of residual PbO is due to the addition of 20% excessive PbO in the ceramic target for compensation of the Pb loss during film deposition. It also showed mixed crystalline orientations of (101) (dominating) and (00*l*) (secondary) for the perovskite PZT phase. It is noted that the predominant film growth plane of (101) is the most densely packed crystalline plane in the perovskite structure and better matches the underlying Pt(111) layer. The polycrystalline grains that had nucleated before annealing were recrystallized into a (001)-preferred orientation during the RTA, under a strong buffering effect of the (001)-oriented LaNiO_3_ layer [9,26]. It is observed that, except for the as-grown film which was highly stressed, all the PZT films showed sharp and high intensity peaks with similar peak positions, indicating a strain relaxation via the high temperature RTA process [9]. On the other hand, the integrated intensity of the (001) PZT XRD peak and its full width at half maximum (FWHM) are shown in the inset of Figure 2 as functions of the RTA annealing time *t*. A decreasing peak intensity with *t* > 5 min indicates a reducing volume of crystallized PZT, which can be ascribed to the proliferation of nanopores in these films. However, the degree of crystallinity showed an increasing trend via a decreasing FWHM. These observations corresponded to a growth of the grains together with nanopores in the PZT films, which are consistent with the SEM results shown in Figure 3.

Figure 3a–d are the cross-sectional SEM images of the annealed PZT films (RTA 2 min, 5 min, 10 min and 15 min). There are two noticeable morphological features that had evolved with an increasing annealing time—the grain shape and nanopores. As shown in Figure 3a,b, the grains are mostly columnar with a small amount of nanopores when the RTA time were at 2 and 5 min [9]. However, the columnar grains recrystallized and coalesced with an increasing annealing time and they became large equiaxed grains in films annealed for 10 and 15 min (Figure 3c–d). Such a microstructure evolution is a classic example of the textbook “structure zone model” [28], in which temperature-induced equiaxial recrystallization and grain growth took place in sputtered films that underwent an extended high temperature process. Furthermore, a substantial amount of nanopores were observed in these films, which were concentrated near the film’s bottom interface with LaNiO_3_ in the RTA 10 min film (Figure 3c) and became evenly distributed in the RTA 15 min film (Figure 3d). The proliferation and re-distribution of nanopores in these films can also be attributed to the recrystallization of the PZT grains into a (001)-preferred orientation, which started from the film’s buffered bottom interface. Such a recrystallization process not only changed the grain shape from columnar to equiaxial, but also drove the residual PbO out and left nanoscale voids/nanopores in the film, leading to the deterioration of the film’s electrical properties. A quantitative EDS analysis showed that the cationic ratio of Pb/(Zr+Ti) decreased with an increasing annealing time, from ~1.12 in the RTA 2 min film to ~1.05 in the RTA 15 min film [9]. This set of data is consistent with the SEM results shown in Figure 3. It is noted that, due to a “damaged” interface by the concentrated nanopores, these defects might have a larger impact on the electrical properties of the PZT RTA 10 min film [29,30]. For example, an applied external electric field might concentrate at or near the interface nanopore region, leading to a lower electric field inside the film bulk. This inhomogeneous field distribution usually corresponds to poor ferroelectric, dielectric and piezoelectric performances [14,15].

**Figure 4:**
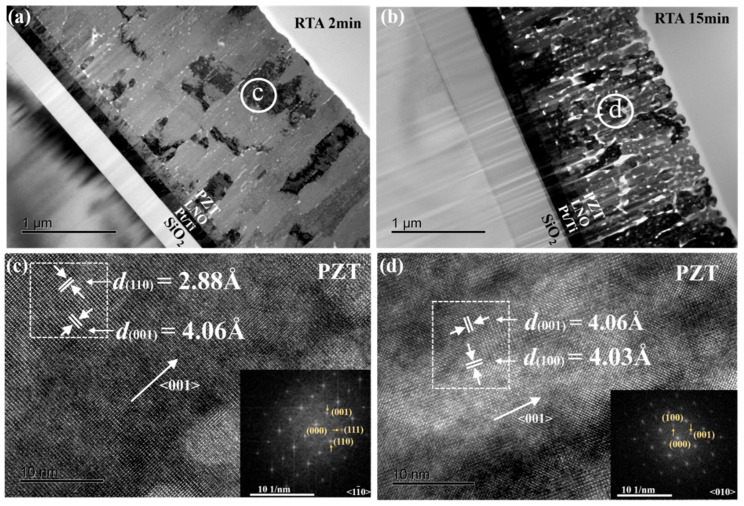
Cross-sectional TEM images of PZT films annealed for (**a**) 2 min and (**b**) 15 min. (**c**,**d**) High-resolution TEM images taken from (**a**,**b**), respectively, insets are selected area electron diffraction patterns (taken from the dashed rectangles) via Fast Fourier Transformation (FFT).

To demonstrate the structural change in the nanoscale, we performed cross-sectional TEM analyses of the two typical PZT films (RTA 2 min and RTA 15 min). As shown in Figure 4a, when the PZT film was annealed for a short time period (2 min), it showed a dense columnar grain morphology with only a small amount of tiny nanopores (~a few tens of nm in diameter). On the other hand, as shown in Figure 4b, when the annealing time was extended (15 min), a large number of bigger nanopores (~30–100 nm in diameter) appeared and were randomly distributed in the whole PZT film. Moreover, volume ratios of nanopores (“porosity”) in the two PZT films with a short (2 min) and a long (15 min) annealing time were estimated by analyzing the color contrast in the two TEM images (Figure 4a,b), where the pores manifested themselves as pure white regions due to a zero resistance against pass of electrons. The porosity of the RTA 2 min and RTA 15 min films were estimated to be 1.0% and 9.9%, respectively. Together with the EDS results reported in Ref. [9] and the SEM images shown in Figure 3, it can be concluded that the removal of residual PbO took place in PZT films that underwent an extended annealing, which left nanopores in the PZT films. In our previous work, we also discussed the removal of residual PbO in PZT films sputter-deposited on (100) Si and annealed via RTA [26,27], which were confirmed by results of quantitative EDS [26] and X-ray photoelectron spectroscopy (XPS) [27].

Additionally, Figure 4c,d are high-resolution TEM images taken from the RTA 2 min and RTA 15 min films, respectively. The inset images are selected area electron diffraction (SAED) patterns taken from the areas encircled by the dashed rectangles via Fast Fourier Transformation (FFT). It is noted that the two TEM samples shown in Figure 4 had different zone axes, <1 −1 0> for the RTA 2 min film and <0 1 0> for the RTA 15 min film. The lattice parameters of the out-of-plane [001] and in-plane [110] directions of the RTA 2 min film were measured to be 4.06 Å and 2.88 Å, respectively, which are close to the bulk values of the PZT 53/47 ceramic (4.03 Å for [001], 2.89 Å for [110]). Similarly, the lattice parameters of the out-of-plane [001] and in-plane [100] directions of the PZT RTA 15 min film matched closely with the bulk values. These data showed that the two films were both close to a relaxed state and had similar lattice parameters. Such an observation is consistent with the XRD patterns shown in Figure 2, i.e., an extended annealing did not vary the average lattice parameters of the PZT films.

**Figure 5:**
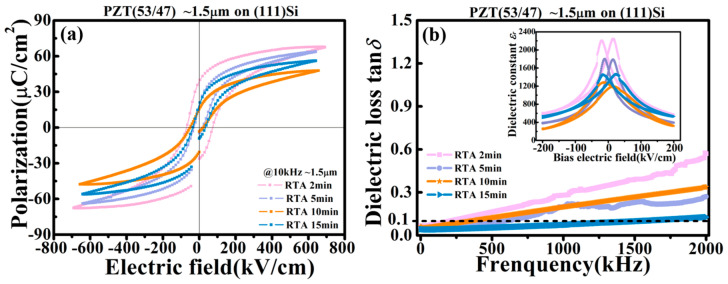
(**a**) Room-temperature ferroelectric P-E loops and (**b**) dielectric losses (tg*δ*) as functions of the measuring frequency of the PZT films deposited on (111) Si and RTA annealed for 2–15 min; inset in (**b**) displays the dielectric constant-bias electric field (*ε_r_*-E) curves of the PZT films.

The room-temperature ferroelectric hysteresis loops (P-E loops) of the PZT films are shown in Figure 5a. The RTA 2 min film displays the highest remnant and saturated polarizations (*P_r_*~39.0 μC/cm^2^, *P_s_*~67.6 μC/cm^2^) and the largest coercive field (*E_c_*~73 kV/cm), when compared with those annealed for a longer time. Furthermore, in the dielectric constant-bias electric field (*ε_r_*-E) curves shown in the inset of Figure 5b, the RTA 2 min film also shows the largest small field dielectric constant *ε_r_* (~1865 @ zero bias field). However, from the dispersion curves of the dielectric loss tangents (tg*δ*-*f*), the RTA 2 min film showed the highest dielectric loss in the full range of the measuring frequency (1 kHz–2 MHz, especially in the high end). On the other hand, the RTA 15 min film showed the opposite trends in its electrical performance, i.e., the lowest polarization values and *E_c_*, as well as the smallest *ε_r_* and tg*δ*. These property data agreed well with our proposed model of the two competing effects on the electrical performance, i.e., reduction in residual PbO (hence a decreased dielectric loss) and proliferation of nanopores (hence lowered electrical polarizations, coercive field and dielectric constant) with an increasing annealing time. The electrical performances of the two films with intermediate annealing times are between those of the RTA 2 min and 15 min ones. The RTA 10 min film, of special note, showed the worst overall performance among the four films, which includes the smallest *P_r_*, *P_s_* and *ε_r_*, and a loss tangent only slightly better than that of the RTA 2 min film. These performance data are well correlated with the microstructure characteristics shown in Figure 3, i.e., a porous coarse grain structure with concentrated nanopores near the film’s bottom interface [29,30]. Such a microstructure is at the transition point of the film’s morphology evolution, based on our analysis of the SEM images.

It is noted that near the low end of the measuring frequency (1 kHz–200 kHz), the dielectric losses of the PZT films showed different yet close numbers, and all are low enough (<0.1) to allow an inverse transverse piezoelectric test aimed for micro-actuator applications. For all the PZT films, the dielectric loss did not vary significantly with an increasing frequency until it enters the sub-Mega and Megahertz range (200 kHz–2 MHz), under which dielectric relaxations from internal charge carriers and defects substantially affect the dielectric properties [31]. In this range, all films except the RTA 15 min one showed a rapidly increasing tan*δ* with the measuring frequency. This behavior can be attributed to their large amount of defects, especially the residual PbO, from an incomplete recrystallization process. The RTA 2 min film, of special note, showed the fastest increase in its dielectric loss with an increasing frequency, which can be ascribed to its highest concentration of residual PbO. Lastly, the RTA 10 min film showed a slightly higher tanδ than the RTA 5 min film, due to its transitioning grain morphology with interface-concentrated nanopores. Overall, based on the structure and property data shown in Figure 2 through Figure 5, our PZT films underwent a short RTA are suited for low-frequency electromechanical applications, while those underwent an extended RTA can be considered for high-frequency applications in microelectronic circuits.

**Figure 6:**
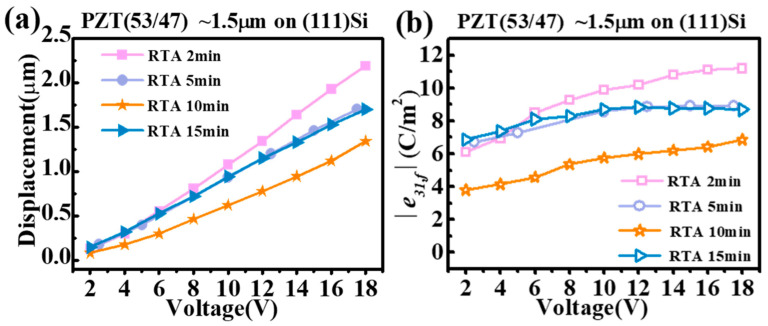
(**a**,**b**) Tip displacements and |*e_31,f_*| transverse piezoelectric coefficients as functions of the applied AC voltage of the PZT film cantilever beams deposited on (111) Si and RTA annealed for 2–15 min.

Figure 6a,b display the tip displacements (δ) and the computed converse transverse piezoelectric coefficients (*e_31,f_*) of the PZT film cantilever beams as functions of an applied external AC voltage (sine wave, 500 Hz) [26,32]. There was no pre-poling step used, and the applied voltage increased monotonically from 2 V to 18 V. It can be seen that all films except the RTA 10 min one showed similar piezoelectric displacement and *e_31,f_* values at a low AC voltage (V ≤ 6 V). This can be explained by a low poling field (~40 kV/cm) which does not fully activate the ferroelectric domains in the films, especially the RTA 2 min film, which has the largest coercive field *E_c_* ~73 kV/cm. However, as the voltage was increased to 8 volts and higher, the RTA 2 min film showed an accelerated increase in the piezoelectric displacement, corresponding to an increasing *e_31,f_*. It was not until the voltage reached 18 volts (~ 120 kV/cm), close to twice as large as the coercive voltage of the RTA 2 min film, that its *e_31,f_* piezoelectric coefficient kept on increasing and left those of the RTA 5 min and RTA 15 min films well behind. The highest *e_31,f_* value achieved was that of the RTA 2 min film at 18 volts, which is about −11.2 C/m^2^ and close to those reported for (001) PZT film grown on the commonly used (100) Si substrate [26,27]. This high piezoelectric performance of the RTA 2 min film is consistent with its (001)-preferred crystalline orientation (Figure 1) with a dense columnar grain structure (very few pores, Figure 3 and Figure 4), as well as its outstanding ferroelectric and dielectric properties (Figure 5). On the other hand, the two films with an extended RTA time, i.e., RTA 5 min and RTA 15 min, showed similar piezoelectric performances with a saturated *e_31,f_* coefficient ~−8.5 C/m^2^ at V ≥ 8 volts. These characteristics are consistent with the proliferation of nanopores in these films, which caused reduced ferroelectric and dielectric performances (Figure 5), as well as a reduced coercive field and hence a lower poling voltage. Lastly, due to a transitioning grain morphology with a “damaged” interface [14,15], the piezoelectric performance of the RTA 10 min film was the poorest among the PZT films, which started at ~−4 C/m^2^ and saturated at ~−6 C/m^2^. It is noted that the *e_31,f_* values obtained by the current method are apparent/average converse e_31,*f*_ piezoelectric coefficients at a given AC electric field/voltage, [33]. The nonlinear piezoelectric responses of a ferroelectric, including a Rayleigh law-type behavior for the small field piezoelectric coefficient, are often the results of extrinsic contributions including the domain wall contribution to the piezoelectric property [34]. In our case of a ferroelectric film, its nonlinear piezoelectric response can be properly investigated by combining an experimental approach that we recently developed [33,35] with the analytical method reported in Ref. [34]. Such an investigation will require superimposing a small signal AC field on a sweeping dc bias field to test the instantaneous piezoelectric coefficient as a function of the dc bias, and a collection of abundant data points under sub-coercive bias fields. The outcome of this investigation will help us achieve a deeper understanding of the structure–property relationship in these PZT films, for example, how the domain wall mobility and density of domain walls vary as a function of the annealing time. Therefore, we plan to carry out a dedicated study in the near future on the piezoelectric responses of these PZT films.

## 4. Conclusions

The microstructure and electrical properties of PZT thick films prepared on (111) Si substrate by a “two-step” method were systematically analyzed. Two competing effects with an increasing annealing time in the RTA step (@ 700 °C), were revealed by comparing the ferroelectric, dielectric and piezoelectric performances of the films, as well as their nanoscale grain morphologies. While the removal of residual PbO certainly helps improve the high-frequency electrical performance, a proliferation of nanopores deteriorates the ferroelectric polarization and dielectric constant of the film, leading to a poorer transverse piezoelectric performance and a smaller *e_31,f_* coefficient. Therefore, the RTA 2 min PZT film, which showed close to bulk values of the ferroelectric polarization and dielectric constant, is better suited for low-frequency piezoelectric applications. Under a bias AC voltage of 18 volts, this film showed a measured *e_31,f_* value ~−11.2 C/m^2^. Lastly, a film morphology change from columnar grains to equiaxial ones was observed in the PZT film with a RTA annealing time of ~10 min at 700 °C. The analyses of the above structure–property relationships in (001)-oriented PZT films integrated on (111) Si will enlighten their applications in Si-based MEMS and microelectronic devices.

In our previous work, PZT films with a similar thickness needed at least 2 min of RTA time at the same temperature to remove surface residual PbO, which is critical for the measurement of reliable electrical properties [27].

## Data Availability

Data available upon reasonable request to author(s).
